# Clinical features, course, and risk factors of infection-associated secondary hemophagocytic lymphohistiocytosis

**DOI:** 10.1007/s15010-025-02559-z

**Published:** 2025-05-27

**Authors:** Michael Ruzicka, Thomas Wimmer, Hans-Joachim Stemmler, Stephanie-Susanne Stecher, Hendrik Schulze-Koops, Fabian Hauck, Marion Subklewe, Michael von Bergwelt-Baildon, Karsten Spiekermann

**Affiliations:** 1 Department of Medicine III, Ludwig Maximilian University (LMU) University Hospital, LMU Munich, Munich, Germany; 2 Department of Medicine II, LMU University Hospital, LMU Munich, Munich, Germany; 3 Department of Medicine IV, LMU University Hospital, LMU Munich, Munich, Germany; 4 Division of Pediatric Immunology and Rheumatology, Department of Pediatrics, Dr. Von Hauner Children’s Hospital, LMU Munich, Munich, Germany; 5 German Cancer Consortium (DKTK), Partner Site Munich, Munich, Germany; 6 Comprehensive Cancer Center Munich (CCC), Munich, Germany; 7 Bavarian Cancer Research Center (BZKF), Munich, Germany

**Keywords:** Hemophagocytic lymphohistiocytosis, Secondary HLH, Infection-associated HLH, Prognostic factors of HLH, Risk factors of HLH

## Abstract

Hemophagocytic lymphohistiocytosis (HLH) is an orphan disease characterized by excessive inflammation and poor outcome. We sought to further characterize clinical features, courses, and risk factors of secondary HLH (sHLH) triggered by infection (iHLH). 28 (43.1%) of 65 adult sHLH cases treated at our hospital from 2012–2024 were infection-associated. iHLH patients were mostly male (71.4%). Infectious agents most frequently detected were EBV (57.1%) and leishmania (14.3%). The median time to diagnosis was 13 [6.0;24.8] days. iHLH patients had a mortality rate of 39.3% (median follow-up time: 735 [336;1140] days), worse survival than patients with autoimmune-triggered (hazard ratio: 3.33 (1.01–11.10), p = 0.049), and better survival than patients with paraneoplastic HLH (hazard ratio: 0.19 (0.10–0.84), p = 0.002). Elevated levels of soluble interleukin-2 receptor (sIL2R; > 6,000 I/U), low thrombocyte counts (< 40 G/l), and a history of malignant disease were associated with adverse outcomes. Protracted time to diagnosis was associated with severe disease courses and with leishmaniosis. Further, sIL2R levels correlated positively with prolonged aPTT and thrombocytopenia, and hypertriglyceridemia with elevated INRs. Patients with an elevated sIL2R:ferritin ratio were more likely to have a history of malignant comorbidities. Taken together, sIL2R, thrombocytopenia, and a history of malignant disease are important prognostic factors of iHLH. Patients with high sIL2R levels or hypertriglyceridemia may be at higher risk of bleeding, and patients with elevated sIL2R:ferritin ratios should be assessed for possible malignant comorbidities. Lastly, increased awareness of the disease and newly emerging pathogens (i.e. leishmania) may shorten the time to diagnosis, and thus reduce severe courses of iHLH.

**Graphical Abstract:**

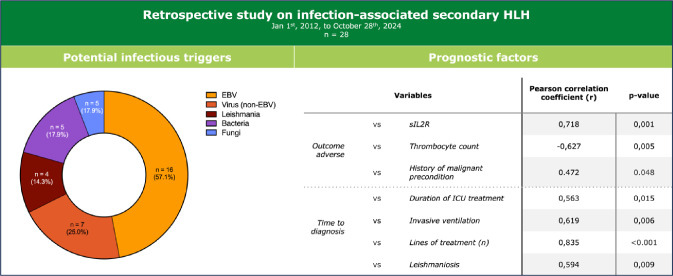

**Supplementary Information:**

The online version contains supplementary material available at 10.1007/s15010-025-02559-z.

## Introduction

Hemophagocytic lymphohistiocytosis (HLH) is a life threatening, rare hyperinflammatory syndrome. Its primary form (pHLH) is commonly found in pediatric patients with underlying genetic alterations and an estimated incidence of 2 × 10^−5^ among children < 15 years old [1]. In contrast, secondary HLH (sHLH) may occur at any age, and shows a lower incidence with a steady increase over the past years (4.2 × 10^−6^ in 2018 [2] or 9.7 × 10^−6^ in 2020 [3]). For sHLH, a variety of medical conditions or therapeutic agents have been described as possible triggers:

•Infectious diseases (iHLH)
•Malignancies
•Autoimmune and autoinflammatory conditions
•Immunosuppression
•Immunotherapy

•Metabolic disorders

A fraction of sHLH cases remains idiopathic.

Clinically, sHLH displays great similarities with sepsis, and the discrimination between the two conditions may be challenging [4]. Diagnostic criteria were established by the HLH Study Group of the Histiocyte Society (HLH-2004 criteria) and include: fever, splenomegaly, cytopenia of ≥ 2 lineages in peripheral blood, hypertriglyceridemia and/or hypofibrinogenemia, elevated serum ferritin, elevated levels of the T cell activation marker soluble interleukin-2 receptor (sIL2R), diminished NK cell activity, and hemophagocytosis in bone marrow (BM), liquor or lymph nodes [5]. Further established diagnostic scores include the HScore, which calculates the probability of an underlying HLH based on clinical and laboratory parameters [6], and the optimized HLH inflammatory index (OHI), which was designed to assess the probability of HLH in patients with malignant comorbidities based on modified sIL2R and ferritin cutoff levels [7, 8].

Treatment recommendations for adult sHLH are largely based on studies conducted in pediatric cohorts [9–11]. Key therapeutic principles are the treatment of underlying triggers (e.g. antineoplastic/-microbial therapy, Rituximab if Epstein-Barr virus (EBV)-associated) and use of immunosuppressive agents (steroids, etoposide, intravenous immunoglobulins (IVIG), interleukin-1 receptor antagonists (i.e. Anakinra), others) [12].

Prognostic risk factors of sHLH have only been assessed in small to medium patient cohorts with heterogeneity regarding the underlying HLH causes. So far, male sex, compromised activated partial prothrombin time (aPTT), and elevated levels of lactate dehydrogenase (LDH), C-reactive protein (CRP) [13] as well as sIL2R [14] have been identified as adverse prognostic factors. To the authors’ knowledge, prognostic factors specific to iHLH in adult patients have not been assessed at the time of writing.

In this study, we sought to further characterize the clinical features and courses of iHLH as well as prognostic factors. We further conducted exploratory correlation analyses to identify clinical constellations with implications for the management of iHLH patients.

## Patients and methods

This study was approved by the institutional ethics committee of the Ludwig Maximilian University (LMU) Munich hospital (project number: 24–0440) and performed in accordance with the 1964 Helsinki Declaration and its later amendments.

### Patient inclusion

Patients ≥ 18 years of age with a confirmed diagnosis of HLH (fulfilling ≥ 5 HLH-2004 criteria [5]) treated at LMU university hospital between January 1st, 2012, and October 28th, 2024, were included. iHLH was diagnosed if an infection was the most likely trigger and an infectious agent was identified. Patients with unknown (lack of documentation) or unclear (various likely) triggers were considered separately.

Of the 65 sHLH patients covered in this study, 42 had been included in a previous work [14]. Here, we mainly focus on a subgroup of iHLH patients to gather new findings specific to this cohort. Of 28 iHLH patients in total, 11 had not been included into the previous work. We assessed further clinical and laboratory parameters compared to the previous study, and extended the follow-up time.

### Detection of infectious pathogens

Viral agents were determined by PCR testing from blood (EDTA and/or serum), nasopharyngeal swabs and/or pharyngeal or bronchial lavage. Bacterial and fungal agents were determined from blood cultures, and aspergillus additionally by galactomannan antigen testing in the serum. Leishmania species were detected via PCR in either bone marrow or spleen tissue. Testing occurred within 7 days of treatment initialization (with the exemption of few leishmania cases, as reflected by longer time to diagnosis) and was followed up as clinically indicated.

### Diagnostic scores

The Hscore [6] and the optimized HLH inflammatory index (OHI) [7, 8] were calculated using laboratory parameters deriving from 24 h within the time point of treatment initialization. Same applies to parameters assessed to evaluate fulfillment of the HLH-2004 criteria.

### Laboratory parameters and lymphocyte subpopulations

Outside of diagnostic scores and criteria, laboratory parameters measured ≥ 72 h prior to or after treatment initialization were assessed. If several measurements during this time were undertaken, the laboratory parameters with the strongest pathological deviation were used. Results of lymphocyte subpopulation analyses were included into the analysis if the measurement was performed < 14 days before or after initiation of HLH treatment.

### Time point of diagnosis and time to diagnosis

If the exact time point of the HLH diagnosis was unknown, the time point of HLH treatment initialization was considered instead. Time to diagnosis was calculated as follows: time between symptom onset (if unavailable: admission to hospital) and time point of diagnosis (if unavailable: HLH treatment initialization).

### Statistical analyses

Statistical tests were performed and resulting figures created using GraphPad Prism Version 10.2.3 and Python 3.12. For continuous variables (e.g. laboratory parameters), median values are reported along with their interquartile ranges in square brackets [quartile 1; quartile 3]. Categorical variables are reported as absolute counts with the respective percentages in round brackets.

Comparisons between laboratory parameters and their reference values were conducted as follows: D’Agostino & Pearson test was used to test Gaussian distribution. If the normality test was passed, one sample two-sided t-test was applied to compare results to the mean of the reference ranges (assuming normal distribution). If the normality test failed, medians of the results and of the respective reference ranges were compared using the nonparametric Wilcoxon signed rank test. If the reference was a cutoff value (i.e. CRP ≤ 0.5 mg/dl), results were tested against the highest value within the reference margin. p-values were adapted for multiple comparisons by Bonferroni correction and considered statistically significant if < 0.05.

Survival data were compared using log-rank test and considered statistically significant at p-values < 0.05. Mantel–Haenszel hazard ratios (HR) are reported alongside their 95% confidence intervals in round brackets. For correlation analysis, binary categorical data were coded as numeric. Correlation was tested using Pearson’s correlation. Results are displayed as Pearson’s correlation coefficients r. Due to the very high number of exploratory pairwise comparisons (> 1,500), p-values of correlation analyses were not adjusted for multiple testing. Instead, key findings of the correlation analysis were validated with additional tests (i.e. Kaplan–Meier survival analysis). p-values were considered statistically significant if < 0.05.

## Results

### sHLH triggers

**Fig. 1:**
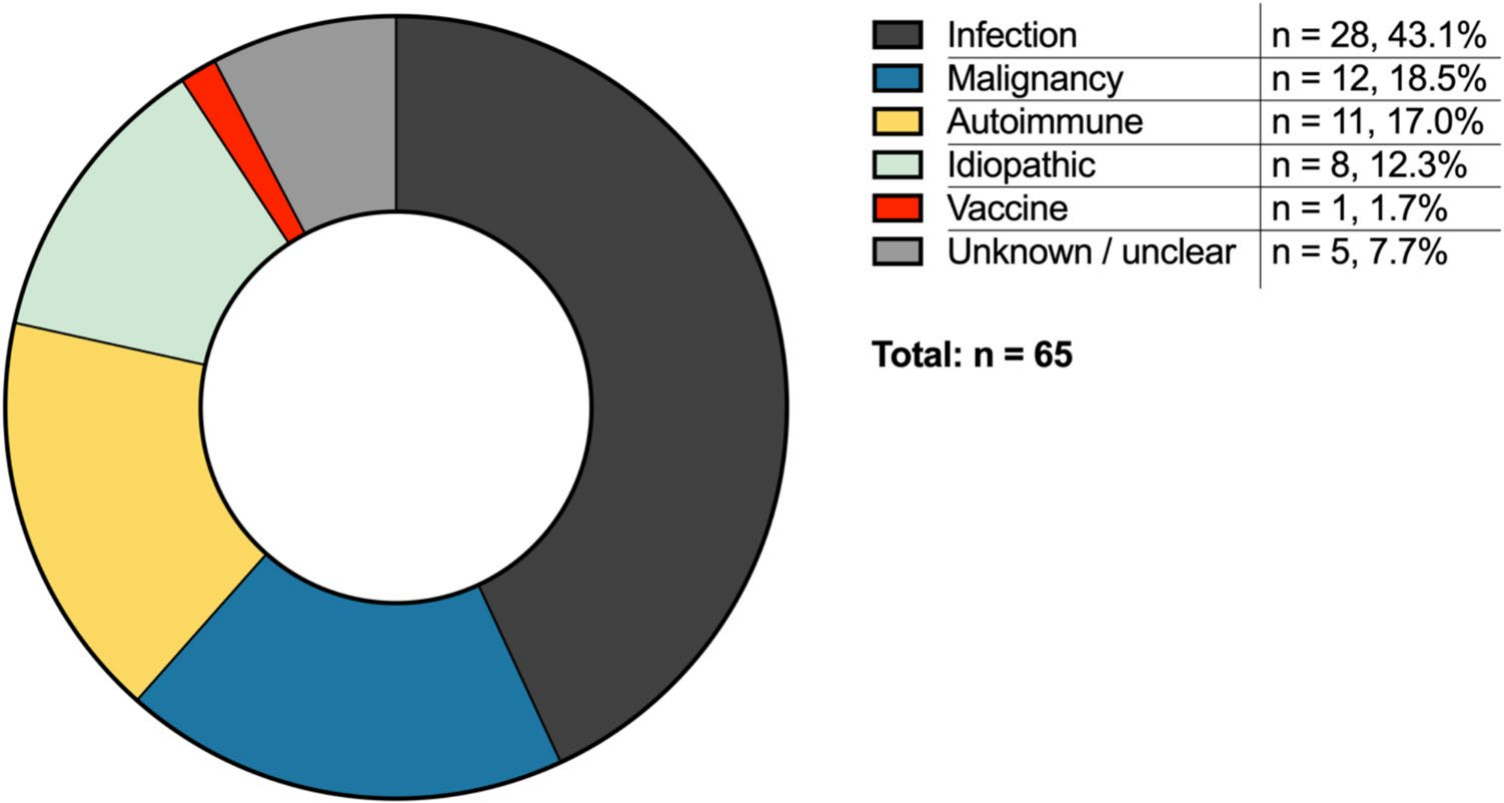
sHLH cases by triggers. sHLH cases treated at LMU university hospital from Jan 1st, 2012, to October 28th, 2024, are depicted based on their respective triggers. Unknown = lack of documentation; unclear = various likely triggers

We identified a total of 65 adult patients who had been treated at the LMU University Hospital Munich with sHLH from 2012–2024. Most of those cases were triggered by infection (n = 28, 43.1%), followed by malignancies (n = 12, 18.5%), autoimmune disorders (n = 11, 17.0%) and vaccines (n = 1, 1.7%; Fig. 1). 8 (12.3%) cases were classified as idiopathic, while the triggers of 5 (7.7%) remained unknown or unclear.

### Characteristics of iHLH patients and infectious triggers

The median age of iHLH patients was 46.0 years [31.8;61.3] and the majority was male (n = 20, 71.4%; Table 1). The median follow-up time was 735 [336;1140] days and the median BMI 25.3 [22.5;28.0] kg/m^2^.

**Table 1:** Demographic data and comorbidities of iHLH patients at the time point of HLH treatment initialization

Patient characteristics	
Gender	
Female (n)	8 (28.6%)
Male (n)	20 (71.4%)
Age (years)	46.0 [31.8; 61.3]
Median follow-up time (days)	735 [336; 1140]
BMI (kg/m^2^)	25.3 [22.5; 28.0]
Type of comorbidity	Detected in n (%) of patients
Psychiatric	3 (10.7)
Somatic	22 (78.6)
Gastrointestinal	11 (39.3)
Cardiovascular	10 (35.7)
Endocrinological	8 (28.6)
Immunosuppressive treatment or condition	8 (28.6)
Organ or bone marrow transplantation	4 (14.3)
Malignant^a^	6 (21.4)
Musculoskeletal	4 (14.3)
Respiratory	2 (7.1)
Other	12 (42.9)

^a^Includes malignant diagnoses in remission and/or with currently no indication for oncologic treatment

Table 1 provides an overview of the comorbidities of iHLH patients. 22 (78.6%) had a history of somatic comorbidities with a median count of n = 2 [1;5]. The most prevalent comorbidities were gastrointestinal (n = 11, 39.3%), cardiovascular (n = 10, 35.7%) and endocrinological (n = 8, 28.6%). 8 (28.6%) patients were on immunosuppressive therapy or had a known immunosuppressive condition. Of those, 2 patients had undergone bone marrow, 1 patient kidney, and 1 patient liver transplantation. 6 patients had a history of malignant disease either in remission and/or without the indication for treatment (including one case of chronic lymphocytic leukemia (CLL) in stage Binet B).

The most frequent infectious agents found in iHLH patients were EBV (n = 16, 57.1%) and leishmania (n = 4, 14.3%). Further viral, bacterial and fungal agents are depicted in Table 2. Of note, in some patients, various infectious pathogens were detected.

**Table 2:** Infectious pathogens detected in iHLH patients

Infectious agent	Detected in n (%) of patients
Viruses	
EBV	16 (57.1)
CMV	2 (7.1)
HSV	2 (7.1)
Parvovirus B19	2 (7.1)
SARS-CoV-2	1 (3.6)
Bacteria	
*Streptococcus agalacticae*	1 (3.6)
*Escheria coli*	1 (3.6)
*Streptococcus pyogenes*	1 (3.6)
*Staphylococcus aureus*	1 (3.6)
*Streptococcus pneumoniae*	1 (3.6)
Fungi	
*Aspergillus fumigatus*	1 (3.6)
*Candida glabrata*	1 (3.6)
Parasites	
*Leishmania*	4 (14.3)

*EBV* Epstein-Barr virus; *CMV* cytomegalovirus; *HSV* herpes simplex virus; *SARS-CoV-2* severe acute respiratory syndrome coronavirus 2

### Time to diagnosis and diagnostic scores

The median time to diagnosis of iHLH patients was 13.0 [6.0;24.8] days. The median Hscore at diagnosis was 225.5 [243;274] (Table 3). 20 (71.4%) iHLH patients fulfilled the criteria for a positive OHI. The median number of fulfilled HLH-2004 criteria at diagnosis was 6 [5;6], the most frequent one being elevated serum levels of ferritin (100% of patients).

**Table 3:** Hscore, OHI, and fulfilled HLH-2004 criteria at the time point of iHLH treatment initialization

Type of score	Median [IQR]
Hscore	225.5 [243; 274]
OHI positive (n of patients)	20 (71.4%)
HLH-2004 criteria (n of fulfilled criteria)	6 [5;6]
**HLH-2004 criteria**	**Fulfilled in n (%) of iHLH patients**^a^
Fever	23 (85.2)
Splenomegaly	23 (88.5)
Cytopenia of ≥ 2 cell types	19 (67.9)
Hypertriglyceridaemia (≥ 265 mg/dl) OR Hypofibrinogenaemia (< 1.5 g/l)	20 (71.4)
Hypertriglyceridaemia	16 (57.1)
Hypofibrinogenaemia	18 (64.3)
Serum ferritin ≥ 500µg/l	28 (100)
sCD25 ≥ 2.400 U/ml	24 (85.7)
Hemophagocytosis in BM	16 (66.7)

^a^Percentages refer to the number of iHLH patients with information on the respective dataset, not necessarily on the total number of iHLH patients

### Laboratory parameters and lymphocyte subpopulations

Table 4 shows blood laboratory parameters of all 28 iHLH patients. Analysis of lymphocyte subpopulations (n = 9 iHLH patients; data not shown) revealed that the absolute lymphocyte count (median: 150 [122.5;790.0] per µl, p < 0.001) and the relative NK cell count (median: 6.7 [2.7;15.6] % of lymphocytes, p = 0.018) were significantly diminished.

**Table 4:** Blood laboratory parameters of iHLH patients at HLH treatment initialization

Blood laboratory parameter	Unit	Median [IQR]	Reference range	p-value^a^
Sodium	mmol/l	134.5 [133.0; 139.5]	135–145	*n.s.*^b^
Creatinine	mg/dl	1.05 [0.73; 2.9]	0.5–1.0	**0.020**^**c**^
Ferritin	ng/ml	14,444.5 [7,144.5; 33,703.75]	15–150	** < 0.003**^**c**^
CRP	mg/dl	12.5 [5.08; 17.59]	≤ 0.5	** < 0.003**^**b**^
PCT	ng/ml	2.6 [0.6; 8.4]	≤ 0.1	** < 0.003**^**c**^
Albumin	g/dl	2.4 [2.0; 2.87]	3.5–5.2	** < 0.003**^**b**^
IgG	g/l	10.9 [8.08; 13.6]	7.00–16.00	*n.s.*^c^
IgA	g/l	2.19 [1.44; 2.93]	0.70–4.00	*n.s.*^c^
IgM	g/l	1.93 [0.57; 2.52]	0.40–2.30	*n.s.*^b^
Bilirubin	mg/dl	2.95 [0.7; 7.75]	≤ 1.2	**0.036**^**c**^
ASAT	U/l	205.5 [76.5; 474.5]	≤ 34	** < 0.003**^**c**^
ALAT	U/l	112 [33.5; 251]	≤ 34	** < 0.003**^**c**^
ASAT:ALAT-ratio	ratio	2.19 [1.51; 3.13]	0.6–0.8	** < 0.003**^**b**^
γGT	U/l	162 [118; 442.5]	≤ 39	** < 0.003**^**c**^
AP	U/l	212.5 [101.25; 384]	35–105	** < 0.003**^**c**^
LDH	U/l	772 [573.25; 1818.5]	≤ 249	** < 0.003**^**c**^
Triglycerides	mg/dl	261.5 [170.75; 384.75]	< 150	** < 0.003**^**c**^
Leukocytes	G/l	2.41 [1.25; 11.2]	4.00–10.40	*n.s.*^c^
Lymphocytes	G/l	0.38 [0.08;0.74]	1.22–3.56	** < 0.003**^**c**^
Hemoglobin	g/dl	7.95 [7; 10.45]	11.5–15.4	** < 0.003**^**c**^
Thrombocytes	G/l	45.5 [25.5; 77.5]	176–391	** < 0.003**^**c**^
Neutrophils	G/l	1.585 [0.65; 5.43]	1.91–7.37	*n.s.*^c^
INR	ratio	1.28 [1.09; 1.5]	0.8–1.2	** < 0.003**^**c**^
aPTT	sec	39.25 [31.75; 49.25]	22–34	** < 0.003**^**c**^
Fibrinogen	mg/dl	185 [118; 287.5]	210–400	**0.017**^**b**^
Antithrombin III	%	79 [47; 82]	83–118	** < 0.003**^**b**^
D-dimer	µg/ml	8.3 [5.56; 21.35]	≤ 0.5	**0.017**^**b**^
IL-6	pg/ml	90.95 [25.75; 427]	≤ 7.0	** < 0.003**^**c**^
sIL2R	U/l	4601.5 [3287.5; 8487.75]	223–710	** < 0.003**^**c**^
sIL2R:ferritin-ratio	ratio	0.29 [0.18; 1.26]		

p-values refer to the deviation from reference values and were calculated as stated in the Patients and Methods section. *n.s.* not significant; *PCT* procalcitonin; *ASAT* aspartate aminotransferase; *ALAT* alanine transaminase; *γGT* gamma-glutamyl transferase; *AP* alkaline phosphatase; *INR* International Normalized Ratio;* IL-6* interleukin-6

^a^p-values were adjusted for multiple comparisons by Bonferroni correction

^b^One sample two-sided t-test

^c^Nonparametric Wilcoxon signed rank test

### Molecular genetic findings

Two iHLH patients were assessed for mutations of the X-linked inhibitor of apoptosis (*XIAP*) gene. HLH focused exome sequencing was conducted in 5 patients. Overall, genetic alterations were detected in 4 of 7 (57.1%) iHLH patients tested (Table 5).

**Table 5:** Molecular genetic testing

Genetic alteration	Associated condition	Detected in n of patients	Age (years) at iHLH diagnosis	Gender	Infectious trigger	Survival status	Follow-up time (days)
hemizygous c.1141 C > T; p.Arg381 ∗	XIAP deficiency	1	19	Male	EBV	alive	1599
hemizygous c.978_1099 del. P.(Cys327 Ter)	XIAP deficiency	1	42	Male	Bacterial	alive	1597
heterozygous STXBP2 Exon 15 c.1298 C > T p.(Ala433 Val) + heterozygous UNC13D Exon 3 c.175G > A p.(Ala59 Thr)	Unclear	1	42	Male	EBV	alive	369
	FHL3						
homozygous c.208G > T; p.Asp70 Tyr	FHL2	1	35	Female	EBV	alive	528

*FHL3* Familial hemophagocytic lymphohistiocytosis 3; *FHL2* Familial hemophagocytic lymphohistiocytosis 2

In two iHLH patients, XIAP deficiency was detected. One of them (male, 19 years old) eventually underwent allogeneic BM transplantation. Other findings include alterations in the genes *STXBP2*, *UNC13D* and *PRF1*. Further information is shown in Table 5. Of note, all patients with underlying genetic alterations were alive at the end of the respective follow-up time.

### Medical treatment of iHLH

All patients (n = 28, 100%) received steroid treatment (either dexamethasone, prednisolone or methylprednisolone, Supplementary Table 1). Besides steroids, the most frequent treatments were IVIG (n = 12, 42.9%), Rituximab and Etoposide (for both: n = 10, 35.7%). Of 16 EBV patients, 9 received Rituximab. The median number of treatment lines was 3.0 [1.8;3.0]. Further details on the treatments as well as the number of treatment lines applied are depicted in Supplementary Table 1.

### ICU and IMC treatment

Of 28 iHLH patients, 18 (64.2%) were admitted to intensive care (ICU) and 9 (32.1%) to intermediate care units (IMC; Table 6). 21 (75.0%) patients were treated in IMC and/or ICU. The median duration of ICU treatment was 5 [4;62] days, of IMC treatment 10 [2;15] days. Further details on ICU/IMC treatment are depicted in Table 6.

**Table 6:** ICU and IMC treatment of iHLH patients

	n (%) of patients	Days (Median + [IQR])
ICU treatment	18 (64.2)	5 [4; 62]
IMC treatment	9 (32.1)	10 [2; 15]
IMC and/or ICU treatment	21 (75.0)	9 [4; 65.8)]
Invasive ventilation	7 (25.0)	50 [14.5; 61]
Non-invasive ventilation	9 (32.1)	5 [3; 8]
Hemodialysis	11 (39.3)	9 [3.5; 32.5]
CytoSorb® treatment	5 (17.9)	2 [2; 3]
vvECMO	1 (3.6)	3 [3; 3]

*vvECMO* venovenous extracorporeal membrane oxygenation

### Overall survival

**Fig. 2:**
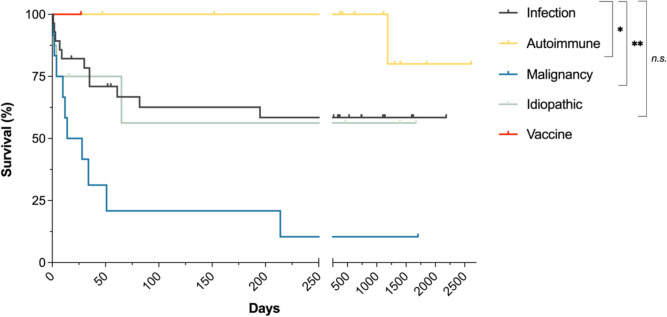
Survival of sHLH patients depending on underlying triggers. Survival data of sHLH patients treated at LMU university hospital from Jan 1st, 2012, to October 28th, 2024, are plotted as Kaplan–Meier survival curves. *n.s.* = not significant (p-value > 0.05); * = p-value ≤ 0.05; ** = p-value ≤ 0.01

Eleven (39.3%) iHLH patients succumbed to the disease during the follow-up time of 735 [336;1140] days. Statistically, survival of patients with iHLH was worse than of patients with autoimmune-triggered (HR = 3.33 (1.01–11.10), p = 0.049, Fig. 2) and better than of patients with malignancy-triggered sHLH (HR = 0.19 (0.10–0.84), p = 0.002). Survival between patients with iHLH and idiopathic sHLH did not differ significantly (HR = 0.85 (0.11–3.27), p = 0.818).

Mortality of patients admitted to ICU was 50.0% compared to 20.0% among patients treated outside of ICU (HR = 2.82 (0.85–9.42), p = 0.091, data not shown). Patients admitted to either IMC and/or ICU showed significantly worse survival than patients who were not (HR = 4.70 (1.31–16.81), p = 0.018, data not shown). Their mortality rates were 52.4% or 0.0%, respectively.

**Fig. 3:**
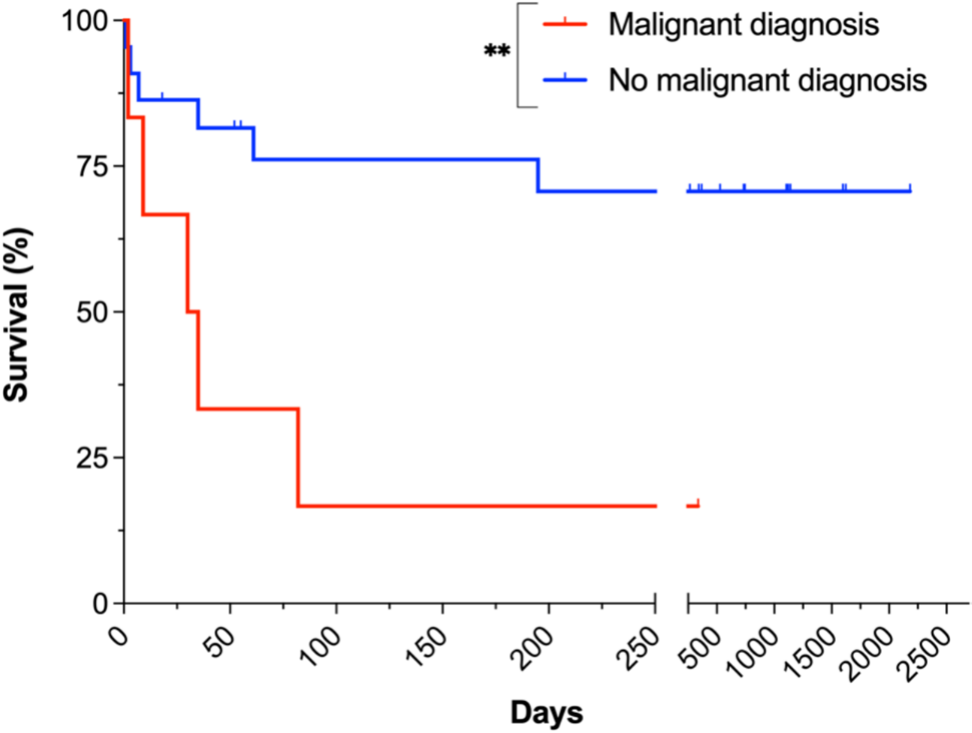
Survival of iHLH patients with and without a history of malignant disease. Kaplan–Meier survival curves of iHLH patients with a history of malignant comorbidities/diagnoses in remission and/or with no indication for oncologic treatment (n = 6) compared to iHLH patients without a history of malignant comorbidities (n = 22). Only patients with infectious HLH triggers were included. ** = p-value ≤ 0.01

Interestingly, patients with a history of malignant disease in remission and/or with no treatment indication showed significantly worse survival than patients without (HR = 8.63 (1.72–43.26), p = 0.009, Fig. 3). Other comorbidities did not impact survival significantly (data not shown).

### Causes of death

Of 10 iHLH patients with lethal outcome, 4 (40%) succumbed to HLH-associated multi-organ failure (MOV) and shock, 4 (40%) to septic shock, and 1 (10%) to pneumonia-associated respiratory failure. The cause of death of one patient discharged into palliative home care is unknown.

### Correlation analysis

**Fig. 4:**
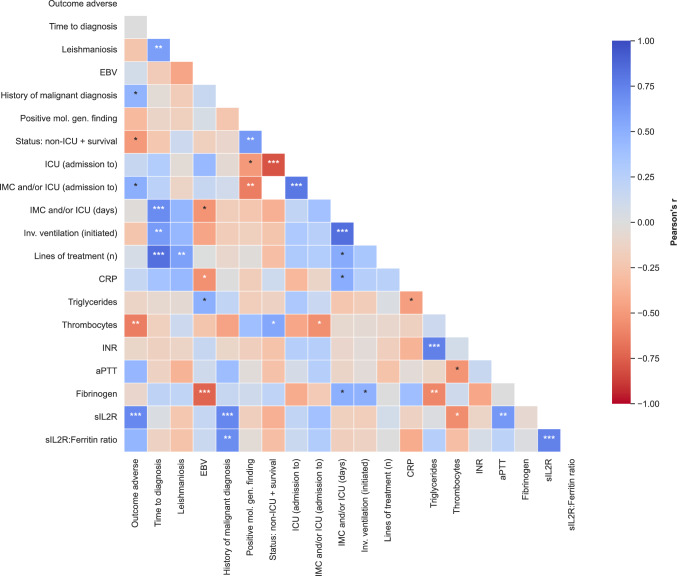
Correlation matrix of iHLH patients’ clinical and laboratory parameters. The parameters assessed include iHLH patients’ demographic, outcome, clinical and laboratory data. The matrix depicts Pearson’s correlation coefficients (r). Positive correlations between the variables are depicted in blue, negative correlations in red, with color intensity reflecting correlation strength (range: r = −1 to + 1). Asterisks indicate statistical significance (* = p ≤ 0.05; ** = p ≤ 0.01; *** = p ≤ 0.001). Only the most relevant findings/parameters from the exploratory correlation analysis are plotted. The full correlation analysis is shown in Supplementary Fig. 1

We conducted a correlation analysis of demographic, outcome, clinical and laboratory data of iHLH patients. In the following, we focus on correlations that are statistically significant, considered clinically relevant, and worth highlighting by the authors (Fig. 4). For other variables of interest please refer to the full correlation matrix (Supplementary Fig. 1).

### Outcome and course of disease

**Fig. 5:**
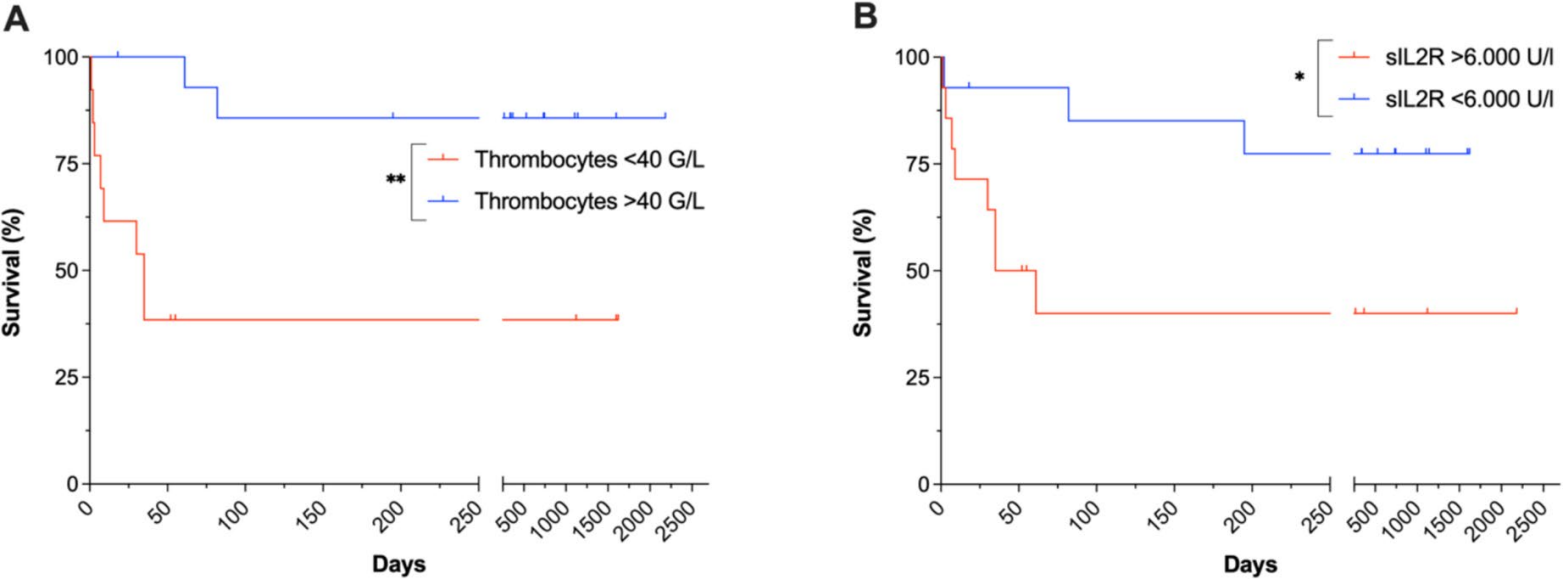
Survival of iHLH patients depending on thrombocyte counts and sIL2R levels. Kaplan–Meier survival curves of iHLH patients with thrombocyte counts (**A**) or sIL2R levels (**B**) below or above the respective cutoff values

Four variables showed statistically significant correlations with adverse outcome (i.e. iHLH-related death): A history of malignant comorbidities either in remission and/or without indication to treat (Pearson’s r = 0.472, p-value = 0.048), admission to IMC and/or ICU (r = 0.500, p-value = 0.035), and elevated levels of sIL2R (r = 0.718, p-value = 0.001) correlated positively, while thrombocyte counts (r = −0.627, p-value = 0.005) correlated negatively with adverse outcomes (Fig. 4). Survival analyses indicate that thrombocytopenia < 40 G/l (HR = 7.99 (2.11–27.23), p-value = 0.002) and sIL2R levels > 6,000 U/l (HR = 3.88 (1.14–13.23), p-value = 0.030) were associated with worse survival (Fig. 5).

To characterize iHLH patients with favorable courses of disease, we assessed all 8 surviving patients who had not been admitted to ICU. They were labeled as “Status: non-ICU + survival” in the correlation analysis (Fig. 4). This group showed positive correlations with molecular genetic findings (r = 0.632, p-value = 0.005) and thrombocyte counts (r = 0.551, p-value = 0.018).

To assess severe disease courses, patients treated in IMC, ICU, or both were clustered into one group (“IMC and/or ICU”). This group correlated inversely with thrombocyte counts (r = −0.551, p-value = 0.018) and molecular genetic findings (r = −0.632, p-value = 0.005). The duration of IMC and/or ICU treatment correlated positively with time to diagnosis (r = 0.696, p-value = 0.001), number of treatment lines (r = 0.528, p-value = 0.024) and CRP levels (r = 0.512, p-value = 0.030).

Lastly, to assess the most severe disease courses exclusively, we considered correlations with ICU admission and invasive ventilation. ICU admission correlated negatively with molecular genetic findings (r = −0.500, p-value = 0.035), and ICU duration positively with time to diagnosis (r = 0.563, p-value = 0.015, Supplementary Fig. 1), as did the need for invasive ventilation (r = 0.619, p-value = 0.006).

### Time to diagnosis

Delayed time to diagnosis was associated with the following: leishmaniosis (r = 0,594, p-value = 0.009, Fig. 4), duration of ICU (r = 0,563, p-value = 0.015) and of IMC and/or ICU treatment (r = 0,696, p-value = 0.001), the need for invasive ventilation (r = 0,619, p-value = 0.006), and the number of treatment lines (r = 0,835, p-value =  < 0.001).

### EBV

EBV correlated positively with serum triglyceride levels (r = 0,497, p-value = 0.036) and negatively with IMC and/or ICU duration (r = −0,528, p-value = 0.024), CRP (r = −0,544, p-value = 0.020) and fibrinogen levels (r = −0,734, p-value = 0.001).

### sIL2R and sIL2R:ferritin ratio

High sIL2R levels were associated with adverse outcomes (r = 0.718, p-value = 0.001), a history of malignant comorbidities (r = 0.722, p-value = 0.001), serum bilirubin levels (r = 0.528, p-value = 0.024), higher aPTT (r = 0.633, p-value = 0.005), and low thrombocyte counts (r = −0.560, p-value = 0.016). The sIL2R:ferritin ratio correlated positively with a history of malignant comorbidities (r = 0.690, p-value = 0.002).

### Coagulation parameters and thrombocyte counts

INRs showed positive correlations with serum triglyceride levels (r = 0.754, p-value < 0.001). aPTT correlated negatively with thrombocyte counts (r = −0.528, p-value = 0.024) and positively with sIL2R levels (see above).

Thrombocyte counts correlated negatively with adverse outcomes, the need for IMC and/or ICU treatment, aPTT as well as sIL2R levels, and positively with favorable courses of disease (see above).

## Discussion

We present a cohort of predominantly male (71.4%) iHLH patients displaying great heterogeneity regarding age as well as number and nature of somatic comorbidities. Previous works confirm that adult sHLH patients are more frequently of male gender and show considerable variety with regard to age [14, 15].

While the association of sHLH with malignancies, chronic infections (e.g. human immunodeficiency virus (HIV) infection) or autoimmune diseases is well established [12, 16], literature on other comorbidities is very scarce. The most frequent comorbidities in our patient cohort were cardiovascular, gastrointestinal and endocrinological. However, we do not assume an overrepresentation of these conditions in our cohort compared to the general population. A significant portion of the presented iHLH patients (n = 8; 28.6%) suffered from immunosuppressive conditions or were on immunosuppressive treatment. Brito-Zerón et al. identified 30 patients who developed HLH following treatment with biological therapies [17]. The authors found infectious triggers in 67% of the cases, making iHLH the most common form of HLH in this cohort. Together, these findings underline that immunosuppressive states pose a relevant risk factor for the development of iHLH, even though no significant correlation between immunosuppression and outcome was detected in our cohort.

EBV was by far the most frequent infectious pathogen, detected in 57.1% of our iHLH patients. It has previously been described to be the most common infectious trigger in both pHLH [18] and sHLH (in up to 44.1% of cases, [15]). The second most frequent infectious agent in our cohort were leishmania, which are known to induce iHLH episodes [19–21]. Recent environmental and climatic transformations promote the spread of leishmaniosis to regions formerly not affected [22]. Thus, it should always be an important differential diagnosis in sHLH patients, even if they do not report travel to endemic areas. The low awareness of leishmania as a potential iHLH trigger is underscored by the positive correlation between leishmania and protracted time to diagnosis.

Blood laboratory values of our iHLH patients indicate hyperinflammation, hepatopathy, impaired liver synthesis and coagulation, and kidney failure as common findings. They further show hypertriglyceridemia, elevated levels of LDH, thrombocytopenia and anemia, lymphopenia, diminished numbers of NK cells, and elevated levels of D-dimer as well as sIL2R. These findings represent common alterations typical for HLH [7, 23, 24].

We found genetic alterations in the genes *XIAP*, *STXBP2*, *UNC13D* and *PRF1* in 4 iHLH patients, all of which had been linked to familial HLH (FHL) [25–28]. The particular *STXBP2* mutation detected in one of our patients (carrying two mutations in total) may be benign and represent a neutral polymorphism, though [26]. Patients in our cohort with HLH-related genetic abnormalities showed milder courses of disease compared to those with no genetic alterations. These results contrast with a study by Bloch et al., who found that an increasing number of HLH-related gene variants was associated with more severe sHLH courses in a cohort of 130 adult patients [15]. Of note, this particular cohort was composed of patients with various HLH triggers, not just iHLH. The number of patients in our study who underwent molecular genetic testing was small. Our findings may thus be subject to selection bias or confounders, must be considered exploratory, and cannot provide definite answers.

Overall, iHLH patients showed better survival than patients with malignancy-associated HLH, and worse outcomes than patients with autoimmune disease-associated HLH. These findings are in line with previous works [14, 15]. Most of our patients were admitted to ICU (n = 18, 64.2%), and 21 (75.0%) were treated in ICU and/or IMC. Together with a mortality rate of 39.3%, this underlines the overall severity of iHLH.

Interestingly, we found that iHLH patients with a history of malignant disease had significantly worse outcomes than patients without. This group also showed higher sIL2R:ferritin ratios. Tsuji et al. identified elevated sIL2R:ferritin ratios as a diagnostic marker specific to lymphoma-associated HLH [29]. Further studies are needed to investigate whether the sIL2R:ferritin ratio may assist in pointing out underlying malignancies or serve as a predictive marker of sHLH in general. Attending physicians should be aware that iHLH patients are at greater risk for adverse outcomes if they have a history of malignant comorbidities, even if those may be in remission or currently with no indication to treat.

High levels of sIL2R (> 6,000 U/l) and thrombocytopenia (< 40 G/l) were associated with adverse outcomes. Thrombocyte counts also correlated with disease severity. Previously, thrombocytopenia has been associated with worse outcomes of sHLH patients in univariate, but not multivariate analyses [13, 14]. Viral infections, visceral leishmaniosis and severe bacterial infections are known to cause thrombocytopenia even in the absence of HLH [30–33]. This may explain the prominent prognostic role of platelet counts in iHLH, where both HLH as well as the infection itself may contribute to and aggravate thrombocytopenia. We further found higher CRP values to positively correlate with more severe courses of disease, while PCT and IL-6 did not.

Of note, we did not find any significant prognostic meaning of the serum ferritin values, HScore, OHI, or the number of fulfilled HLH-2004 criteria in the correlation analysis. These findings are in line with previous publications [13, 14] and allow the conclusion that ferritin and the scores mentioned show their value in diagnosis, but not as prognostic tools in iHLH patients.

Patients with longer time to diagnosis had more severe disease courses, were more likely to need invasive mechanical ventilation, and received more treatment lines. These observations highlight the need for education of medical personnel about this condition to prevent severe disease courses caused by delayed diagnosis of HLH.

High sIL2R levels were associated with compromised aPTT and thrombocytopenia, and hypertriglyceridemia with elevated INRs. Thus, iHLH patients with high sIL2R levels and/or hypertriglyceridemia may be particularly prone to bleeding. As all HLH patients, they should be thoroughly monitored for bleeding signs.

Limitations of our study include its retrospective character and relatively low number of patients. Due to the low number of analyses performed, lymphocyte subpopulations were not included into the correlation matrix, neither were laboratory parameters with missing values for > 3 patients. Genetic testing was only performed in 7 patients. Thus, all conclusions, including prognostic factors and other findings from the correlation analysis, must be considered with caution and validated in larger cohorts. Lastly, we did not draw any conclusions from the medical treatments applied as this study was performed retrospectively and in a non-randomized manner.

As stated before, a fraction of patients had already been assessed in a previous study of our group [14]. Here, we focused on the subgroup of iHLH to identify disease characteristics and prognostic factors specific to infectious HLH triggers. High sIL2R levels had already been described as a risk factor in sHLH [14], and our data confirm the transferability of these findings to the subgroup of iHLH, further identifying thrombocytopenia and a history of malignant disease as adverse prognostic markers. Beyond, the association of CRP with severe disease courses, the frequency of various infectious triggers, the adverse impact of protracted time to diagnosis, and the detailed depiction of clinical/ICU courses provide additional insights beyond the previous study. The extension of the median follow-up time from 288 to 735 days enables more accurate survival analyses.

Taken together, sIL2R, thrombocytopenia, and a history of malignant disease appear to be important prognostic factors of iHLH. Elevated sIL2R:ferritin ratios may be indicative of malignant comorbidities. Increased awareness of the disease and newly emerging pathogens (i.e. leishmania) may shorten the time to diagnosis, and thus reduce severe courses of iHLH. We encourage future studies to investigate prognostic factors of sHLH patients with various triggers. Stratification of sHLH patients based on prognostic scores may eventually enable controlled therapeutic trials with treatment intensity adapted to patients’ individual risk.

## Supplementary Information

Below is the link to the electronic supplementary material.

Supplementary file1 (PDF 577 KB)

## Data Availability

All original data will be made available upon reasonable request to the corresponding author (michael.ruzicka@med.uni-muenchen.de).
