# Supplementary material for: Clinical features, course, and risk factors of infection-associated secondary hemophagocytic lymphohistiocytosis
Source: Infection. 2025 May 27;53(5):2135–47. doi: 10.1007/s15010-025-02559-z (PMC12460370; doi:10.1007/s15010-025-02559-z)
Supplement: Supplementary file 1 — Supplementary file1 (PDF 577 KB) [file 15010_2025_2559_MOESM1_ESM.pdf]

Supplementary Materials

Article Title:

Clinical features, course, and risk factors of infection-associated secondary hemophagocytic lymphohistiocytosis

Journal:

Infection

Authors:

Michael Ruzicka, Thomas Wimmer, Hans-Joachim Stemmler, Stephanie-Susanne Stecher, Hendrik Schulze-Koops, Fabian Hauck, Marion Subklewe, Michael von Bergwelt-Baildon, Karsten Spiekermann

Corresponding author:

Dr. med. Michael Ruzicka

LMU University Hospital

Department of Medicine III, LMU Munich

Marchioninistrasse 15

81377 Munich, Germany

Email: michael.ruzicka@med.uni-muenchen.de

Type of treatment	n (%) of patients
Glucocorticoids	28 (100)
IVIG	12 (42.9)
Rituximab	10 (35.7)
Etoposide	10 (35.7)
Ciclosporine	5 (17.9)
Ruxolitinib	3 (10.1)
Anakinra	3 (10.1)
Cyclophosphamide	1 (3.6)
Plasmapheresis	2 (7.1)
EBV-specific CTLs	1 (3.6)
Allogeneic bone marrow transplantation	1 (3.6)
**Lines of treatment (n)**	**n (%) of patients**
1 (glucocorticoids only)	7 (25.0)
2	6 (21.4)
3	10 (35.7)
4	3 (10.7)
5	0 (0.0)
6	0 (0.0)
7	1 (3.6)
8	1 (3.6)

Supplementary Table 1. Treatment of iHLH patients

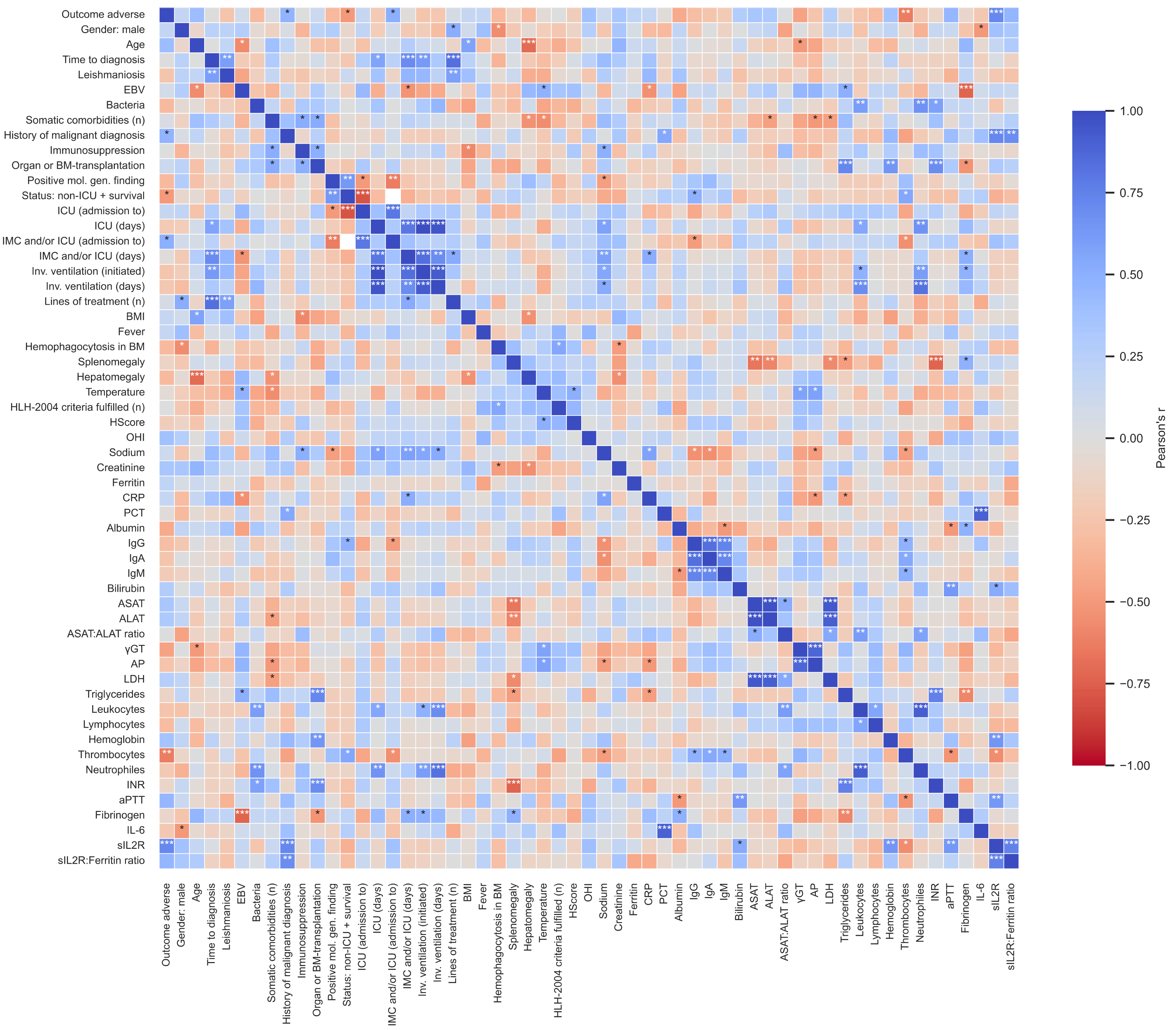

Supplementary figure 1. Full correlation matrix of clinical and laboratory parameters of iHLH patients. The parameters assessed include iHLH patients' demographic, outcome, clinical and laboratory data. Pearson's correlation coefficients (r) between all variables are displayed. Positive correlations are indicated in blue, negative correlations in red, with color intensity reflecting correlation strength (range: r = −1 to +1). Asterisks indicate statistical significance (* = p ≤ 0.05; ** = p ≤ 0.01; *** = p ≤ 0.001).
